# NO-mediated dormancy release of *Avena fatua* caryopses is associated with decrease in abscisic acid sensitivity, content and ABA/GA_s_ ratios

**DOI:** 10.1007/s00425-023-04117-z

**Published:** 2023-04-22

**Authors:** Jan Kępczyński, Agata Wójcik, Michał Dziurka

**Affiliations:** 1 Institute of Biology, University of Szczecin, Wąska 13, 71-415 Szczecin, Poland; 2 Institute of Plant Physiology, Polish Academy of Sciences, Niezapominajek 21, 20-239 Krakow, Poland

**Keywords:** Abscisic acid, After ripening, *Avena fatua*, Dormancy, Gibberellins, Nitric oxide

## Abstract

**Main conclusion:**

NO releases caryopsis dormancy in *Avena fatua*, the effect being dependent on the level of dormancy. The NO effect involves also the reduction of caryopsis sensitivity to ABA and to a decrease in the ABA to GA_s_ ratio due to a decrease in ABA levels and the lack of effect on GA_s_ levels before germination is completed.

**Abstract:**

Nitric oxide (NO) from various donors (i.e. SNP, GSNO and acidified KNO_2_), applied to dry caryopses or during initial germination, released primary dormancy in caryopses. Dormancy in caryopses was gradually lost during dry storage (after-ripening) at 25 °C, enabling germination at 20 °C in the dark. The after-ripening effect is associated with a decrease in NO required for germination. In addition, NO decreased the sensitivity of dormant caryopses to exogenous abscisic acid (ABA) and decreased the embryos’ ABA content before germination was completed. However, NO did not affect the content of bioactive gibberellins (GA_s_) from non-13-hydroxylation (GA_4_, GA_7_) and 13-hydroxylation (GA_1_, GA_3_, GA_6._) pathways. Paclobutrazol (PAC), commonly regarded as a GA_s_ biosynthesis inhibitor, counteracted the dormancy-releasing effect of NO and did not affect the GA_s_ level; however, it increased the ABA content in embryos before germination was completed. Ascorbic acid, sodium benzoate and tiron, scavengers of reactive oxygen species (ROS), reduced the stimulatory effect of NO on caryopsis germination. This work provides new insight on the participation of NO in releasing *A. fatua* caryopses dormancy and on the relationship of NO with endogenous ABA and GA_s_.

**Supplementary Information:**

The online version contains supplementary material available at 10.1007/s00425-023-04117-z.

## Introduction

Harvested viable seeds unable to germinate under favourable conditions are commonly regarded as primarily dormant (Bewley et al. 2013). For germination to begin, seed dormancy must be removed; thus, non-dormant seeds can complete germination after they have been placed under suitable species-specific conditions. Among phytohormones, abscisic acid (ABA) is widely recognised as an agent crucial for the induction and maintenance of seed dormancy; a higher ABA content is often associated with a deep dormancy (Frey et al. 2012). The ABA level (Rodríguez et al. 2009; Liu et al. 2013; Matilla et al. 2015; Sano and Marion-Poll 2021) was most often reduced by dormancy-releasing factors, e.g. stratification or after-ripening. While ABA is regarded as a principal factor responsible for dormancy control, the overall balance between ABA and GA_s_, including their contents and signalling, is considered to be mainly responsible for the establishment, maintenance, and release of dormancy (Bewley et al. 2013; Liu et al. 2013; Matilla et al. 2015; Rodriguez et al. 2015; Benech-Arnold and Rodriguez 2018; Carrillo-Barral et al. 2020; Sano and Marion-Poll 2021). Other hormones, e.g. ethylene, auxins, jasmonates and brassinosteroids, have also been shown to be involved in the regulation of seed dormancy state (Kępczyński and Kępczyńska 1997; Feurtado et al. 2007; Corbineau et al. 2014; Ali et al. 2022).

Caryopses of *A. fatua*, a very important annual weed infesting major cereal crops in many regions of the world, including Poland, are an interesting model system on which to study the mechanism of dormancy release (Simpson 1990; Kępczyński 2018, 2023). Primary dormancy in caryopses can be removed by after-ripening, GA_3_, karrikin 1 (KAR_1_) and hydrogen peroxide (Kępczyński 2018, 2023). Endogenous GA_s_ and ethylene were demonstrated to be required for *A. fatua* caryopsis dormancy release by KAR_1_ (Kępczyński 2018). The stimulatory effect of KAR_1_ is associated with non-transcriptional and transcriptional activation of 1-aminocyclopropane-1-carboxylic acid (ACC) synthase and ACC oxidase enzymes, respectively, and with modulation of ethylene sensitivity through control of the ethylene receptors synthesis (Ruduś et al. 2019). ABA was found to play an important role in caryopsis dormancy of *A. fatua*; the dormancy release by KAR_1_ involved reduction of the ABA content in embryos, coleorhiza and radicle (Kępczyński et al. 2021). Previously, coleorhiza-enforced seed dormancy was proposed as a mechanism controlling germination in *A. fatua* and other grasses (Holloway et al. 2020).

NO, an uncharged, gaseous lipophilic free radical can regulate seed dormancy and germination in several dicot plant species (Bethke et al. 2007; Arc et al. 2013; Matilla et al. 2015; Singorelli and Considine 2018; Kumar et al. 2021). Various NO donors, such as sodium nitroprusside (SNP), S-nitroso-*N*-acetylpenicillamine (SNAP), S-nitrosoglutathione (GSNO) or acidified KNO_2_ were found to promote dormancy release in apple (Gniazdowska et al. 2007), *Arabidopsis* (Bethke et al. 2004), lettuce (Belgini and Lamattina 2000) and redroot pigweed (Kępczyński and Sznigir 2014) seeds. Thus, different NO donors have often been used in experiments with seeds of various plant species, aimed at elucidating the role of NO. The role of NO in inducing germination of dormant seeds has been studied mainly in dicots, whereas no sufficient information is available in monocots. Very early studies showed nitrogen dioxide to remove dormancy, probably via NO, in red rice seeds (Cohn and Castle 1984). The stimulatory effect of SNP in monocots has been described in a few papers only, one focussing on *H. vulgare* grain SNP treatment (Bethke et al. 2004) and the other two dealing with seeds of prairie grasses *Panicum virgatum*, *Andropogon gerardii* and *Sorghastrum nutants* (Sarath et al. 2006) as well as with germination of the cereal crop *Triticum aestivum* (Jacobsen et al. 2013). In addition, Sarath et al. (2006) reported on the effect of NO released from acidified KNO_2_ on germination of *P. virgatum*. Similarly, there is only limited information on the interaction between NO and plant hormones. This information can be found in studies addressing the relationship between NO and ABA or methyl jasmonate in dormancy regulation of seeds of *P. virgatum* (Sarath et al. 2006) and *T. aestivum* (Jacobsen et al. 2013). There is no information on the contribution of NO to the caryopsis dormancy regulation in monocot weeds, including *A. fatua*. It is only known that non-dormant *A. fatua* embryos produce NO and its level is lowered by 2-(4-carboxyphenyl)-4,4,5-tetramethylimidazoline-1-oxyl-3-oxide (cPTIO) (Kępczyński and Cembrowska-Lech 2018).

Thus, the present study was aimed at explaining the relationship between NO and after-ripening, ABA and gibberellins (GA_s_) in releasing the *A. fatua* caryopsis dormancy. The objective was pursued by determining effects of vapours of various NO donors: SNP, GSNO and acidified KNO_2_, on germination of dormant caryopses, and by following the response of caryopses after-ripened for various periods of time to vapours of acidified KNO_2_. The interaction between NO and ABA was examined by determining germination of caryopses treated with vapours of acidified KNO_2_ in the presence of ABA, and by exploring the influence of acidified KNO_2_ used after various periods of germination on the ABA content in embryos. The linkage between NO and GA_s_ was examined by determining the GA_s_ contents in embryos treated with vapours of acidified KNO_2_, and effects of paclobutrazol (PAC), a GA_s_ biosynthesis inhibitor, on germination of caryopses treated with vapours of acidified KNO_2_ and PAC treatment of caryopses on ABA and GA_s_ contents in embryos. The relationship between NO and reactive oxygen species (ROS), known to participate in *A. fatua* caryopsis dormancy release (Kępczyński 2018), was examined using ROS scavengers in combination with NO. The results should provide new data on the role of NO in releasing caryopsis dormancy and its involvement in regulating the endogenous ABA and GA_s_ contents before germination is completed.

## Materials and methods

*Avena fatua* (wild oat) spikelets were collected in 2011 and 2015. The florets-containing spikelets were dried at room temperature to constant moisture of caryopses of ca. 11% (7 days) and stored at − 20° C until required. To remove dormancy, air-dried florets from the 2015 harvest were stored at ambient humidity in the dark at 25 °C for various periods, up to 16 weeks. Only the caryopses (dehulled florets) or embryos were used in the experiments.

### Treatment of air-dried caryopses with NO donors

Dormant or after-ripened (for various periods) dry caryopses (25 in 3 replicates each) were placed in open 6-cm diameter Petri dishes. Three Petri dishes with dry caryopses and one open 6 cm Petri dish with water (control) or a donor solution were placed in a 19 cm diameter Petri dish which was sealed with 3 layers of Parafilm and kept for various periods of time at 20 °C and at 120 µmol photons m^−2^ s^−1^ light or in the dark. Subsequently, untreated (control) and treated caryopses were transferred to Petri dishes with filter paper moistened with 1.5 ml water. Three Petri dishes with caryopses were placed in a 19-cm diameter Petri dish along with one 6 cm diameter Petri dish with water, and kept in the dark for up to 7 days.

### Treatment of caryopses with NO donors during initial germination

Dry dormant caryopses (25 in 3 replicates each) were placed in open 6-cm diameter Petri dishes on a single layer of filter paper moistened with 1.5 ml distilled water, ABA (10^–6^, 10^–5^, 3 × 10^–5^ 10^–4^ M), PAC (10^–6^, 10^–5^, 10^–4^ M) or ascorbic acid, sodium benzoate, or tiron (10^–3^ M) solutions. Three open Petri dishes with caryopses on water and one open 6-cm diameter Petri dish with water (control) or a donor solution were placed in a 19 cm diameter Petri dish. Three open Petri dishes with caryopses in a compound solution tested together with one open 6-cm diameter Petri dish with water or a donor solution were placed in a 19 cm diameter Petri dish. The 19 cm Petri dishes were sealed with 3 layers of Parafilm and kept for various periods of time at 20 °C at 120 µmol photons m^−2^ s^−1^ light or in the dark. After an appropriate period of treatment, the Petri dish with the donor solution was replaced with a Petri dish with water and kept at 20 °C in the dark for up to 7 days.

### Applied NO donors: SNP, GSNO and acidified KNO_2_

#### SNP

3 ml of SNP solutions each (10^–3^, 3 × 10^–3^, 10^–2^ M) were used for treating of air-dried caryopses for 5 or 24 h in the light.

#### GSNO

A 3 ml mixture of 2 × 10^–4^ or 10^−3^ M GSNO and 10^–3^ M GSH was used for treating air-dried caryopses in the light for 24 h or during the first 24 h of their germination in water in the light.

##### KNO_2_ solution acidified with H_2_SO_4_

A 5 ml mixture of 10^–3^ or 2 × 10^−3^ KNO_2_, 10^–1^ M H_2_SO_4_, 10^–1^ M KI and 1.4 × 10^–1^ M K_2_SO_4_ was used for (i) a 3 h treatment of dry caryopses after-ripened for various periods, or (ii) for 3 h during initial germination in water or ROS scavenger solutions in the dark.

##### KNO_2_ solution acidified with HCl

A 5 ml mixture of 10^–2^ KNO_2_ and HCl 2 × 10^−1^ M was applied for 3 h during initial germination in water or in ABA solutions, or during the initial germination for 24 h in water or PAC solutions. In the experiment aimed at determining ABA and GA_s_ contents, the donor was applied during 3 h after 15, 21 or 33 h of germination.

### Determination of caryopsis germination

The caryopses were regarded as germinated when the radicle protruding through the coleorhiza was longer than 1 mm. The Petri dishes were handled under green light (0.5 µmol m^−2^ s^−1^) which does not affect germination.

### Determination of ABA and GA_s_ contents

Dormant caryopses (25 in 3 replicates each) were incubated in 6-cm diameter Petri dishes on filter paper moistened with 1.5 ml water and placed in a 19 cm diameter Petri dish, along with a 6 cm diameter Petri dish containing water, for 15, 21 and 33 h at 20 °C in the dark. After an appropriate time, three 6 cm diameter Petri dishes with caryopses were transferred, for 3 h, to a 19 cm diameter Petri dish containing a 6 cm diameter Petri dish with water (control) or acidified KNO_2_ (a 5 ml mixture 10^–2^ M KNO_2_ and 2 × 10^–1^ M HCl); the dishes were kept at 20 °C in the dark. In one experiment, dormant caryopses (25 in 3 replicates each) were incubated in 6 cm diameter Petri dishes on filter paper moistened with 1.5 ml water or 10^–4^ M PAC and were placed along with a 6 cm diameter Petri dish with water in a 19 cm diameter Petri dish, and were kept for 30 h at 20 °C in the dark. When incubation was completed, the embryos were isolated. ABA and GA_s_ were analysed as described by Dziurka et al. (2019), with some modifications. After the material was lyophilized, 10 mg samples were pulverised with zirconia beads. A stable-isotope-labelled internal standard mixture was added to each sample. Following extraction and cleaning up the samples on hybrid SPE cartridges (BondElut Plexa PCX, Agilent, Sanat Clara, CA, USA), the hormone contents were measured in the MRM mode of Agilent Infinity 1260 and with 6410 Triple Quad LC/MS (Agilent). An Ascentis Express RP-Amide analytical column (2.7 μm, 2.1 mm × 75 mm; Supelco, Bellefonte, PA, USA) was used. The quantification was based on calibration curves obtained for pure standards and recoveries of internal standards. The phytohormone standards were obtained from Olchemim (Olomouc, Czech Republic).

### Statistical treatment

The mean ± standard deviation (SD) of three replicates was calculated and plotted as bar diagrams. Significance of differences between the means was tested using one- or two-way analysis of variance (ANOVA; Statistica for Windows v. 10.0, Stat-Soft Inc., Tulsa, OK, USA). Duncan’s multiple range test was used to identify significantly different (*P* ≤ 0.05) mean values. Similar results were obtained in two independent experiments with caryopses germination.

## Results

### Effects of SNP and GSNO vapours on germination of dormant caryopses

**Fig.1:**
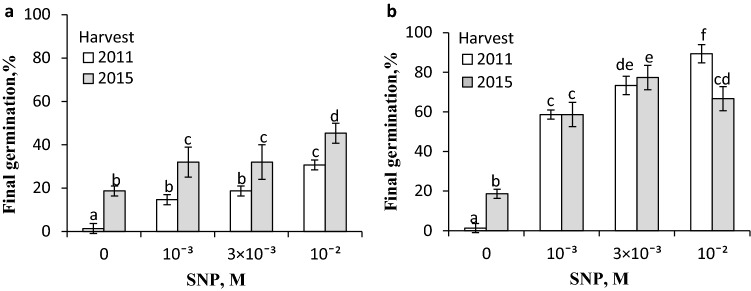
Effect of vapours released from SNP on germination of *A. fatua* caryopses. Dormant dry caryopses from the 2011 and 2015 harvests were incubated in the presence of vapours from SNP for 5 h (**a**) or 24 h (**b**). Vertical bars indicate ± SD. One-way ANOVA with Duncan’s post hoc test was used to test for significance of differences. Means denoted by different letters (**a**–**f**) are significantly different (*P *˂ 0.05, *n* = 3)

**Fig. 2:**
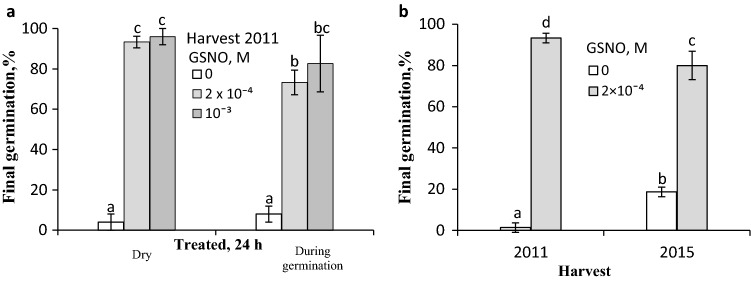
Effect of vapours released from GSNO on germination of *A. fatua* caryopses. Dormant dry caryopses from the 2011 harvest were treated for 24 h with vapours from GSNO or during initial 24 h of germination in water (**a**). Dormant dry caryopses from the 2011 and 2015 harvest were treated for 24 h with vapours from GSNO (**b**).Vertical bars indicate ± SD. One-way ANOVA with Duncan’s post hoc test was used to test for significance of differences. Means denoted by different letters (**a**–**d**) are significantly different (*P *˂ 0.05; *n* = 3)

The caryopses harvested in 2011 and 2015 were either unable to germinate or their germination amounted to ca. 20%, respectively (Fig. 1). A 5-h exposure of dry caryopses to SNP vapours increased germination of caryopses from both harvests, the highest effect being obtained when the highest concentration was applied (Fig. 1a). Thus, at 10^–2^ M, 30 or 40% of caryopses germinated, depending on the harvest year. The magnitude of the SNP effect was evident when the treatment was extended to 24 h (Fig. 1b). At 3 × 10^–3^ and 10^–2^ M SNP, 70 and 90% of the caryopses harvested in 2011 germinated, respectively. At the same SNP concentrations, ca. 70 and 80% of the caryopses from 2015 harvest germinated. Effects of GSNO vapours applied for 24 h to dry caryopses or during the initial 24 h of germination of caryopses from the 2011 harvest were comparable. GSNO at concentrations of 2 × 10^–4^ and 10^–3^ M resulted in germination of almost all the previously treated dry caryopses (Fig. 2a). When the caryopses were treated during germination, the stimulatory effect was somewhat lower at the lower GSNO concentration. Likewise, dry caryopses from the 2015 harvest responded distinctly to GSNO, 80% of the caryopses being able to germinate (Fig. 2b).

### Effects of acidified KNO_2_ vapours on germination of caryopses after various periods of florets after-ripening

**Fig. 3:**
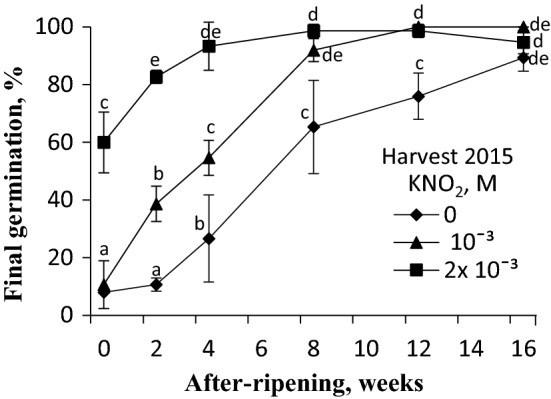
Effect of vapours released from acidified KNO_2_ on germination of *A. fatua* caryopses harvested in 2015 after various periods of after-ripening. Dry caryopses after various periods of after-ripening were treated, for 3 h, with vapours from 10^–3^ and 2 × 10^–3^ M KNO_2_ acidified with H_2_ SO_4_. Vertical bars indicate ± SD. One-way ANOVA with Duncan’s post hoc test was used to test for significance of differences. Means denoted by different letters (**a**–**e**) are significantly different (*P *˂ 0.05; *n* = 3)

The germination percentage was increased following dry after-ripening, the effect being intensified as the storage duration was extended (Fig. 3). After-ripening of florets for 16 weeks resulted in an almost complete germination of caryopses. A 3 h exposure of dry caryopses to vapours from acidified 10^–3^ M KNO_2_ resulted in about 40% germination of the caryopses stored for 2 weeks, whereas the untreated caryopses germinated at a percentage (10%) similar to that observed in the non-after-ripened caryopses. The germination percentage of caryopses treated with vapours released by 10^–3^ M KNO_2_ increased as after-ripening duration was extended: an almost complete germination was found just after 8 weeks of after-ripening. When a higher concentration of the donor, 2 × 10^–3^ M, was used, ca. 60% of non-after-ripened caryopses germinated and a shorter after-ripening duration was sufficient for a complete germination.

### Effects of acidified KNO_2_ vapours on germination of caryopses in the presence of exogenous ABA and on the ABA and GA_s_ contents in embryos

**Fig. 4:**
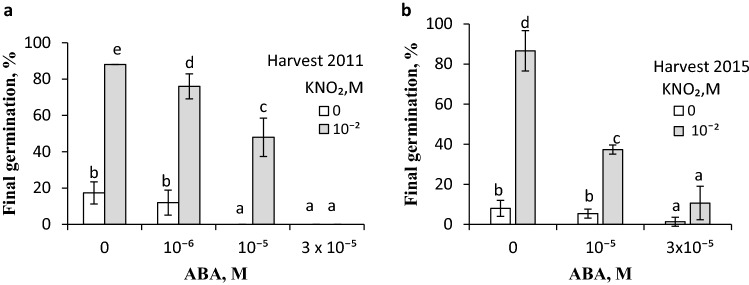
Effect of vapours released from acidified KNO_2_ on germination of *A. fatua* caryopses in the presence of ABA. Dormant caryopses from the 2011 (**a**) and 2015 (**b**) harvests were treated during initial 3 h of germination in water or ABA solutions with vapours from KNO_2_ solution acidified with HCl. Vertical bars indicate ± SD. One-way ANOVA with Duncan’s post hoc test was used to test for significance of differences. Means denoted by different letters (**a**–**e**) are significantly different (*P *˂ 0.05, *n* = 3)

**Fig. 5:**
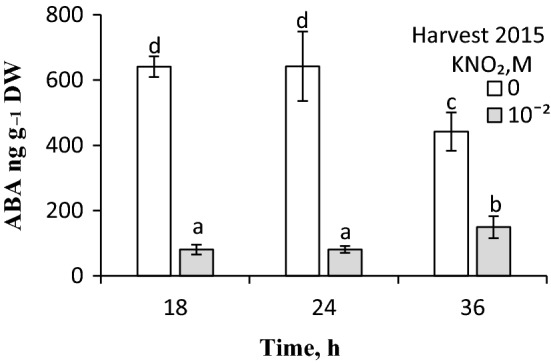
Effect of vapours released from acidified KNO_2_ on the ABA content in embryos of *A. fatua* caryopses after 18, 24 and 36 h of germination. Dormant caryopses from the 2015 harvest were treated for 3 h with vapours from KNO_2_ solution acidified with HCl after 15, 21 and 33 h of germination. Changes in the ABA content in embryos from dormant caryopses during germination in water were described previously (Kępczyński et al. 2021). Vertical bars indicate ± SD. One-way ANOVA with Duncan’s post hoc test was used to test for significance of differences. Means denoted by different letters (**a**–**d**) are significantly different (*P *˂ 0.05, *n* = 3)

Vapours released from acidified KNO_2_ solution applied for 3 h during initial germination markedly enhanced the process; germination was completed in ca. 90% caryopses from the 2011 harvest, whereas the untreated caryopses germinated in ca. 20% (Fig. 4a). When applied at concentrations higher than 10^–6^ M, ABA completely prevented germination of dormant caryopses. When vapours from acidified KNO_2_ were applied during the initial 3 h of germination, 75 and 50% of the caryopses germinated, despite the presence of ABA at 10^–6^ or 10^–5^ M, respectively. The vapours were not able to stimulate germination when ABA was used at a concentration of 3 × 10^–5^ M. Application of the donor also counteracted the ABA effect on germination of caryopses from the 2015 harvest (Fig. 4b). Moreover, vapours applied to caryopses for 3 h between 15 and 18 h or between 21 and 24 h of germination resulted in the embryos ABA content being 8 times lower than in embryos from untreated caryopses (Fig. 5). The ABA content in embryos from caryopses exposed to vapours for 3 h (between germination hour 33 and 36) was 3 times lower than the content in embryos from untreated caryopses. All the bioactive GA_s_ from non-13-hydroxylation pathways, i.e. GA_4_ and GA_7_, and those from 13-hydroxylation pathways, i.e. GA_1_, GA_3_, and GA_6_ were also identified (Table S1). The content of polar bioactive GA_s_ (GA_1_ + GA_3_ + GA_6_) was several times higher than the content of GA_4_ + GA_7_. The contents of individual GA_s_ during germination did not differ. Vapours from acidified KNO_2_ did not change the level of GA_s_.

### Effect of PAC on germination of caryopses treated with vapours from acidified KNO_2_ and on the ABA and GA_s_ contents in embryos

**Fig. 6:**
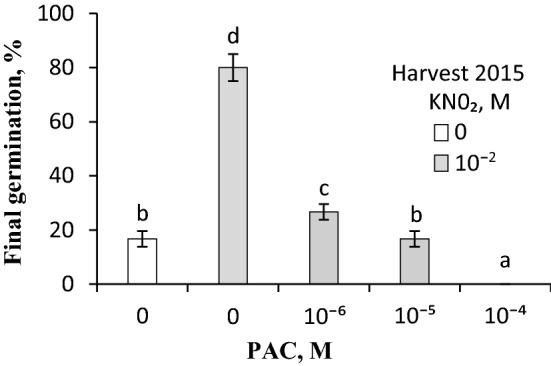
Effect of vapours released from acidified KNO_2_ on germination of *A. fatua* caryopses in the presence of PAC. Dormant caryopses from the 2015 harvest were treated with vapours from KNO_2_ solution acidified with HCl during the initial 24 h of germination in PAC solutions. Vertical bars indicate ± SD. One-way ANOVA with Duncan’s post hoc test was used to test for significance of differences. Means denoted by different letters (**a**–**d**) are significantly different (*P *˂ 0.05, *n* = 3)

Caryopses exposed to vapours of acidified KNO_2_ for the initial 24 h of germination germinated in ca. 80%, compared to ca. 15% germination of untreated caryopses (Fig. 6). PAC strongly inhibited germination of vapour-treated caryopses, the effect being related to the PAC concentration. At 10^–6^ and 10^–5^ M, as little as ca. 25 and 15% caryopses germinated, respectively, despite the vapour exposure. The treatment with 10^–4^ M PAC completely inhibited germination of the vapour-treated caryopses. The ABA content was determined in embryos isolated from caryopses incubated in water or in a PAC solution for 30 h (Table 1). The ABA content in embryos was increased due to the PAC treatment. However, PAC did not affect the level of GA_s_ originating from either non-13-hydroxylation or 13-hydroxylation pathways (Table S2).

**Table 1:** Effect of PAC on the ABA content in embryos of *A. fatua* caryopses after 30 h of germination. Dormant caryopses from the 2015 harvest were used. Vertical bars indicate ± SD. One-way ANOVA with Duncan’s post hoc test was used to test for significance of differences. Means denoted by different letters (**a**–**b**) are significantly different (*P *˂ 0.05, *n* = 3)

PAC, M	ABA ng g^−1^DW
0	554.67 ± 3.21^a^
10^–4^	730.33 ± 106.78^b^

### Effects of vapours from acidified KNO_2_ on caryopsis germination in the presence of free-radical scavengers

**Fig. 7:**
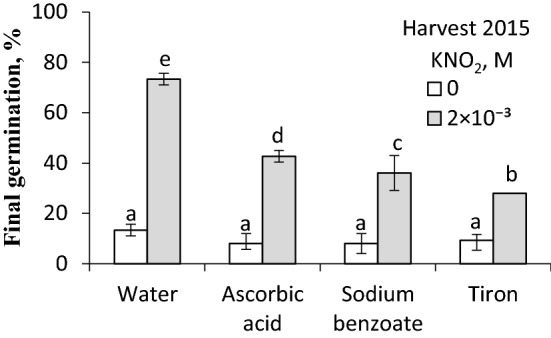
Effect of vapours released from acidified KNO_2_ on germination of *A. fatua* caryopses in the presence of ROS scavengers. Dormant caryopses from the 2015 harvest were treated for initial 3 h of germination in the presence of scavengers with vapours from KNO_2_ solution acidified with H_2_ SO_4_. The scavengers were used at 10^–3^ M concentration. The scavengers were used at 10^–3^ M concentration. Vertical bars indicate ± SD. One-way ANOVA with Duncan’s post hoc test was used to test for significance of differences. Means denoted by different letters (**a**–**e**) are significantly different (*P *˂ 0.05, *n* = 3)

Effects of free-radical scavengers, ascorbic acid, sodium benzoate and tiron, on germination of dormant caryopses exposed for 3 h to vapours of acidified KNO_2_ during initial germination were investigated to explore possible interactions between NO and reactive oxygen species. None of the ROS scavengers tested affected germination of dormant caryopses (Fig. 7). However, ascorbic acid and sodium benzoate reduced germination in the vapour-treated caryopses; 35–40% of them germinated. The highest inhibitory effect (germination of as little as ca. 25% of caryopses) was attributed to tiron.

## Discussion

### Release of caryopsis dormancy by NO and by after-ripening

### NO

In studies on the role of NO in germination of dicot seeds (Bethke et al. 2007; Arc et al. 2013) as well as those of barley (Bethke et al. 2004) and wheat (Jacobsen et al. 2013), SNP was often used as an NO donor either by germination the seeds in solution or by exposing the germinating seeds to SNP vapours. These vapours (Fig. 1) as well as those of other NO donors such as KNO_2_ and GSNO (Yamasaki 2000; Cantrel et al. 2010), applied to dry caryopses or during initial germination, stimulated germination of dormant *A. fatua* caryopses from both harvests (Figs. 2, 4), supporting the notion that the stimulation occurs on account of NO generation, which—like in seeds of other species (Kumar et al. 2021)—plays an important role in dormancy release. Regardless of the type of donor and application manner, NO released dormancy in *A. fatua* caryopses as effectively as GA_3_ (Kępczyński 2018; Holloway et al. 2020) or KAR_1_ did (Kępczyński 2018).

### After-ripening

Dormancy of *A. fatua* caryopses can be also released during dry after-ripening of florets (Kępczyński et al. 2021; Fig. 3); caryopses are then able to complete germination. The after-ripening mediated transition from dormant to non-dormant caryopses involved a reduced sensitivity to ABA (Kępczyński et al. 2021). In contrast, after-ripening increased the sensitivity to NO (Fig. 3) as well as to GA_3_ and KAR_1_ (Kępczyński 2018). Thus, it reduced the need for those regulators. Moreover, dormancy release by floret after-ripening was associated with a reduced ABA level in embryos from after-ripened caryopses before germination was completed (Kępczyński et al. 2021). Different responses to NO of dormant and after-ripened caryopses are probably related to different ABA contents in embryos from these caryopses. Dormancy release by after-ripening and cold stratification of wheat grains was also associated with reduced ABA levels in germinating seeds (Tuttle et al. 2015). Thus, after-ripening released dormancy in dry seeds and brought about a reduction in the ABA content before germination was completed. Other experiments with wheat showed that after-ripening did not alter the seed ABA content before and during germination (Liu et al. 2013). Thus, although the ABA level reduction is a response common in seeds of several species due to after-ripening (Matilla et al. 2015), it might be considered whether the hormone is the primary effector of dormancy release in the case of some seeds. In turn, it was demonstrated that the ABA content in the coleorhiza played a key role in controlling dormancy and germination of barley (Barrero et al. 2009). Recently, it has been also postulated that that the coleorhiza-enforced dormancy in caryopses of *A. fatua* (Holloway et al. 2020). After-ripening, also KAR_1_ decreased the ABA content in the coleorhiza before germination was completed (Kępczyński et al. 2021). ABA inhibited radicle emergence after-ripened caryopses *A. fatua* more strongly than coleorhiza emergence (Holloway et al. 2020; Kępczyński et al. 2021).

### The relationship between NO and ABA

To examine the relationship between NO and ABA, ABA concentrations versus one concentration of NO were used, like in experiments using dormant or non-dormant *Arabidopsis* seed (Bethke et al. 2006; Liu et al 2009) and dormant apple embryos (Gniazdowska et al. 2007). Treating dormant caryopses with ABA showed the hormone to deepen the level of dormancy, expressed as inhibition of germination (Fig. 4), like in dormant apple embryos (Gniazdowska et al. 2007). NO removed dormancy in apple embryos (Gniazdowska et al. 2007) and *Arabidopsis* seeds (Bethke et al. 2006) and counteracted the inhibitory effect of ABA, which allowed to conclude that NO reduced the sensitivity to ABA. NO was also able to dampen the sensitivity of *A. fatua* caryopses to ABA (Fig. 4), indicating a similar relationship between these factors in dicot seeds and monocot caryopses. The effect of NO on germination of dormant caryopses involves also an ABA content reduction, the effect being weaker when NO was applied after a longer germination period (Fig. 5). This is in agreement with previous data from experiments on dicot seeds, e.g. *Arabidopsis*, showing the inducement of dormancy release by NO to be associated with a decreasing ABA level (Liu et al. 2009, 2010) and demonstrating that NO-induced dormancy release in apple embryos was associated with down-regulation of genes responsible for the ABA synthesis (Andryka-Dudek et al. 2019). Thus, the dormancy-releasing effect of NO, both in dicot seeds and monocot caryopses, is associated with a reduction of the ABA content. Possibly, an NO-induced decrease in the ABA level in *A. fatua* embryos (Fig. 5) is a result of ABA degradation to phaseic acid, as shown for the caryopsis response to KAR_1_ (Cembrowska-Lech and Kępczyński 2016). In dormant seeds of *Arabidopsis*, NO was found to induce dormancy release by decreasing the ABA content, which was associated with an increased expression of CYP707A2 encoding ABA 8-hydroxylase responsible for the conversion of ABA to phaseic acid (Liu et al. 2009).

### The NO relationship with GA_s_ and ABA

The fact that NO was found to be unable to change the contents of GA_s_, both from non-13-hydroxylation and 13-hydroxylation pathways in *A. fatua* embryos before caryopsis germination was completed (Table S1), may suggest that the stimulatory effect of NO does not require an increased GA_s_ content. It was reported that the dormancy release in barley by another factor, after-ripening, is not related to the GA_s_ level change (Jacobsen et al. 2002; Barrero et al. 2009). On the other hand, it was suggested that dormancy removal by after-ripening in wheat seeds was associated with an increasing GA_s_ level during germination (Liu et al. 2013). Based on an experiment with *Arabidopsis* (Bethke et al. 2007), it was suggested that NO stimulates germination by increasing the GA_s_ level and reducing the ABA-imposed dormancy (Sanz et al. 2015; Kumar et al. 2021).

PAC, a triazole which blocks the GA_s_ biosynthesis by inhibiting the oxidation of ent-kaurene (Desta and Amare 2021), strongly counteracted the stimulatory effect of NO (Fig. 6) and KAR_1_, another dormancy release inductor (Kępczyński 2018; Ruduś et al. 2019), suggesting a possibility that endogenous GA_s_ are required for dormancy release by these compounds. However, PAC was also found to increase the expression of key biosynthetic genes, *GA3ox* and *GA20ox*, during soybean seed germination, the expression being considered as a compensating mechanism in response to PAC (Gazara et al. 2019). Moreover, it was reported that PAC down-regulated the expression not only of the gene encoding ent-kaurene oxidase but also of *GA2ox* encoding enzymes responsible for degradation of bioactive GA_s_ (Nagar et al. 2021). Thus, it seems possible that the GA_s_ level in embryos from PAC-treated caryopses (Table S2) could remain unchanged despite the inhibition of biosynthesis due to their inhibited degradation. PAC and other triazoles can also increase the ABA content in two ways: by increasing its synthesis, when ent-kaurene oxidation is inhibited or by deactivating it by inhibition of the ABA 8-hydroxylase activity (Yamaguchi et al. 2007; Desta and Amare 2021). Although PAC did not affect the GA_s_ level (Table S2), it did increase the ABA content (Table 1). Taking into account the stimulatory effect of NO and the inhibitory effect of PAC on germination and on reducing or increasing the ABA content, respectively, and also in view of the absence of any effect on the GA_s_ contents, it can be assumed that the response to NO does not require any increase in the latter, but a reduction of the ABA level is probably necessary. Presumably, a reduction of the ABA content renders the concentration of GA_s_ sufficient for germination of dormant caryopses. The ABA catabolism is assumed to be a crucial step in the transition between dormancy and germination (Ali et al. 2022). The discussion of the role of ABA and GA_s_ leads to the conclusion that dormancy, in the case of cereals, is for the most part controlled by the ABA content and the caryopsis sensitivity to the hormone (Kumar et al. 2013). Thus, the stimulatory effect of NO on the *A. fatua* caryopsis germination could probably involve a decrease of the ABA/GA_s_ ratio due to the decreasing ABA content. Likewise, the ABA/GA_s_ ratio is assumed to be playing a central role in the control of dormancy and germination in both dicot and cereal seeds (Tuan et al. 2018; Ali et al. 2022).

### The interaction between NO and ROS scavengers

It was previously demonstrated that ROS, e.g. H_2_O_2_, and also aminotriazole (a catalase activity inhibitor) (Amory et al. 1992) induce germination of dormant caryopses and reduce the ABA content in *A. fatua* embryos (Kępczyński 2023). H_2_O_2_ was also found to induce germination of barley seeds, which was associated with a reduction of the ABA content through the ABA catabolism (Ishibashi et al. 2017). Ascorbic acid, responsible for removing H_2_O_2_, counteracted the dormancy release effects of GA_3_ and KAR_1_ (Cembrowska-Lech and Kępczyński 2016) as well as that of NO (Fig. 7). Also tiron, known to remove the superoxide anion (Taiwo 2008), and sodium benzoate, a hydroxyl radical scavenger (Dey et al. 2021) decreased the stimulatory effect of NO (Fig. 7), suggesting that some level of ROS is required for caryopses to respond to NO. H_2_O_2_ was found to stimulate germination and NO production, and to reverse the inhibitory effect of ABA in warm-season C_4_-grasses (Sarath et al. 2007). Dormancy release in *A. fatua* caryopses by GA_3_ and KAR_1_ was related to an increasing content of H_2_O_2_ and activities of superoxide dismutase and catalase, indicating that the ROS homeostasis is probably required for germination of these caryopses (Kępczyński 2023). Thus, the cross-talk between NO, ABA and ROS in releasing dormancy and in stimulating germination should be taken into account. Regarding the ROS involvement, the idea of an “oxidative window” should be referred to with respect to establishing a ROS level appropriate for seeds to germinate. If the level is too low, the seeds are dormant, whereas a too high a level results in damages (Bailey et al. 2008).

To sum up, NO plays an important role as a dormancy release inductor in *A. fatua* caryopses. Various donors of NO can be used to remove dormancy in both air-dried caryopses or those undergoing initial germination. After-ripening, which released dormancy of caryopses, intensified their response to NO. Effects of NO include also a reduction in the ABA content in embryos, without affecting the GA_s_ contents, before the caryopsis germination is completed. The reversal of the stimulatory effect of NO by PAC was related to a reduction of the ABA content without a change in the GA_s_ levels. The dormancy release by NO in *A. fatua* caryopses, and possibly in some other grasses, involves a decrease of the ABA/GA_s_ ratio by a reduction in the ABA content in embryos during early stages of germination, and a decreased sensitivity to the hormone. In addition, the stimulatory effect of NO on dormancy release involves presumably ROS.

#### *Author contribution statement*

JK conceived and designed the research, interpreted results and wrote the manuscript. AW conducted the physiological experiments. MD carried out the analysis of hormones. All the authors read, reviewed and approved the manuscript.

## Supplementary Information

Below is the link to the electronic supplementary material.

Supplementary file1 (DOCX 18 KB)

Supplementary file2 (DOCX 15 KB)

## Data Availability

The data sets generated and/or analysed during the current study are available from the corresponding author on reasonable request.

## Abbreviations

GSNO: S-Nitrosoglutathione
KAR_1_: Karrikin 1
NO: Nitric oxide
PAC: Paclobutrazol
ROS: Reactive oxygen species
SNP: Sodium nitroprusside
