# Supplementary material for: NO-mediated dormancy release of Avena fatua caryopses is associated with decrease in abscisic acid sensitivity, content and ABA/GAs ratios
Source: Planta. 2023 Apr 22;257(6):101. doi: 10.1007/s00425-023-04117-z (PMC10122620; doi:10.1007/s00425-023-04117-z)
Supplement: Supplementary file 1 — Supplementary file1 (DOCX 18 KB) [file 425_2023_4117_MOESM1_ESM.docx]

| **GA_s_ ng g ^-1^DW** | | **Time, h** | | | |
| --- | --- | --- | --- | --- | --- |
|  |  | 18 | | 36 | |
|  |  | H_2_O | 10^-2^ M KNO_2_ | H_2_O | 10^-2^ M KNO_2_ |
| **GA_1_** | 460.35 ±30.69^a^ | | 456.65 ± 27.24^a^ | 428.52± 48.35^a^ | 383.6 ± 67.39^a^ |
| **GA_3_** | 76.47 ± 19.29^a^ | | 49.17 ± 12^a^ | 48.45 ± 10.92^a^ | 61.28 ± 4.81^a^ |
| **GA_4_** | 53.93 ±15.11^a^ | | 44.97±9.37^a^ | 42.98±7.77^a^ | 37.78±6.66^a^ |
| **GA_6_** | 259.76 ±27.41^a^ | | 284.09 ± 38.10^a^ | 286.78 ± 60.55^a^ | 226.29 ± 15.32^a^ |
| **GA_7_** | 12.78±1.72^a^ | | 13.0±1.63^a^ | 15.22±2.03^a^ | 12.43±1.36^a^ |

Table S1 Effects of vapours released from acidified KNO_2_  on GA_s_ contents in embryos of *A. fatua* caryopses after 18 and 36 h of germination. Dormant caryopses from the 2015 harvest were treated for 3 h with vapours released from KNO_2_ (10 ^-2^ M) solution acidified with HCl after 15 and 33h of germination.

**NO**-**mediated dormancy release of *Avena fatua* caryopses is associated with decrease in abscisic acid sensitivity, content and ABA/GA_s_ ratios**

**Jan Kępczyński^1^, Agata Wójcik^1^, Michał Dziurka^2^**

**^1^Institute of Biology, University of Szczecin, Wąska 13, 71-415 Szczecin, Poland**

**^2^Polish Academy of Sciences, Institute of Plant Physiology, Niezapominajek 21, 20-239 Krakow, Poland**

**Corresponding author Jan Kępczyński**

[**jan.kepczynski@usz.edu.pl**](mailto:jan.kepczynski@usz.edu.pl)**;** [**jankepcz@wp.pl**](mailto:jankepcz@wp.pl)
