# Supplementary material for: NO-mediated dormancy release of Avena fatua caryopses is associated with decrease in abscisic acid sensitivity, content and ABA/GAs ratios
Source: Planta. 2023 Apr 22;257(6):101. doi: 10.1007/s00425-023-04117-z (PMC10122620; doi:10.1007/s00425-023-04117-z)
Supplement: Supplementary file 2 — Supplementary file2 (DOCX 15 KB) [file 425_2023_4117_MOESM2_ESM.docx]

| **PAC, M** | **GA_s_ ng g ^-1^DW** | | | | |
| --- | --- | --- | --- | --- | --- |
|  | **GA_1_** | **GA_3_** | **GA_4_** | **GA_6_** | **GA_7_** |
| **0** | 672±20.66^a^ | 89.33±10.59^a^ | 34.66±5.68^a^ | 112.66±24.11^a^ | 9±0^a^ |
| **10^-4^** | 724.666 ±195.15^a^ | 68±17.77^a^ | 40.33±10.21^a^ | 113.33±13.61^a^ | 12.33±2.51^a^ |

Table S2 Effects of PAC on the GA_s_ contents in embryos of *A. fatua* caryopses after 30 h germination. Dormant caryopses from the 2015 harvest were used.

**NO**-**mediated dormancy release of *Avena fatua* caryopses is associated with decrease in abscisic acid sensitivity, content and ABA/GA_s_ ratios**

**Jan Kępczyński^1^, Agata Wójcik^1^, Michał Dziurka^2^**

**^1^Institute of Biology, University of Szczecin, Wąska 13, 71-415 Szczecin, Poland**

**^2^Polish Academy of Sciences, Institute of Plant Physiology, Niezapominajek 21, 20-239 Krakow, Poland**

**Corresponding author Jan Kępczyński**

[**jan.kepczynski@usz.edu.pl**](mailto:jan.kepczynski@usz.edu.pl)**;** [**jankepcz@wp.pl**](mailto:jankepcz@wp.pl)
